# Sustainable development of a GCP-compliant clinical trials platform in Africa: the Malaria Clinical Trials Alliance perspective

**DOI:** 10.1186/1475-2875-9-103

**Published:** 2010-04-20

**Authors:** Bernhards R Ogutu, Rita Baiden, Diadier Diallo, Peter G Smith, Fred N Binka

**Affiliations:** 1 Malaria Clinical Trials Alliance, INDEPTH Network, 11 Mensah Wood Road, East Legon, PO Box KD 213, Kanda, Accra Ghana; 2 London School of Hygiene & Tropical Medicine, Keppel St, London WC1E 7HT, UK

## Abstract

**Background:**

The Malaria Clinical Trials Alliance (MCTA), a programme of INDEPTH network of demographic surveillance centres, was launched in 2006 with two broad objectives: to facilitate the timely development of a network of centres in Africa with the capacity to conduct clinical trials of malaria vaccines and drugs under conditions of good clinical practice (GCP); and to support, strengthen and mentor the centres in the network to facilitate their progression towards self-sustaining clinical research centres.

**Case description:**

Sixteen research centres in 10 African malaria-endemic countries were selected that were already working with the Malaria Vaccine Initiative (MVI) or the Medicines for Malaria Venture (MMV). All centres were visited to assess their requirements for research capacity development through infrastructure strengthening and training. Support provided by MCTA included: laboratory and facility refurbishment; workshops on GCP, malaria diagnosis, strategic management and media training; and training to support staff to undertake accreditation examinations of the Association of Clinical Research Professionals (ACRP). Short attachments to other network centres were also supported to facilitate sharing practices within the Alliance. MCTA also played a key role in the creation of the African Media & Malaria Research Network (AMMREN), which aims to promote interaction between researchers and the media for appropriate publicity and media reporting of research and developments on malaria, including drug and vaccine trials.

**Conclusion:**

In three years, MCTA strengthened 13 centres to perform GCP-compliant drug and vaccine trials, including 11 centres that form the backbone of a large phase III malaria vaccine trial. MCTA activities have demonstrated that centres can be brought up to GCP compliance on this time scale, but the costs are substantial and there is a need for further support of other centres to meet the growing demand for clinical trial capacity. The MCTA experience also indicates that capacity development in clinical trials is best carried out in the context of preparation for specific trials. In this regard MCTA centres involved in the phase III malaria vaccine trial were, on average, more successful at consolidating the training and infrastructure support than those centres focussing only on drug trials.

## Background

About one million African children die of malaria each year [1]. Advances in molecular biology and biotechnology have brought an increased focus on the development of new drugs, vaccines, insecticides and diagnostic tools that can be deployed to reduce the burden of disease and ultimately to eradicate malaria. The discovery and development of malaria control tools has been promoted through the formation of several international public-private partnerships. These include the Malaria Vaccine Initiative (MVI), the Medicines for Malaria Venture (MMV), the European Malaria Vaccine Initiative (EMVI), the Foundation for Innovative New Diagnostics (FIND), the African Malaria Network Trust (AMANET), the Drugs for Neglected Diseases initiative (DNDi) and the institute for One World Health (iOWH). This has led to the development of a relatively rich product development portfolio over the last two decades. It is essential, however, that the efficacy and effectiveness of new malaria control tools are evaluated in the disease endemic areas [2] and there has been growing recognition of the urgent need to build and strengthen human and infrastructure capacity in Africa to conduct rigorous evaluation trials and studies. This led to the creation of the Multilateral Initiative on Malaria (MIM) and, more recently, the European & Developing Countries Clinical Trials Partnership (EDCTP). However, with few exceptions, relatively little was done either to support the refurbishment of the laboratories and clinics or to develop clinical trial capacity among staff associated with the trial field sites for intervention studies. Rather than developing local capacity, there was a tendency to use external contract research organizations (CROs) to provide the necessary expertise for trial conduct and relatively little was left behind at a research centre after the completion of a trial. Typically, CROs secured the rights to conduct clinical trials, and put African scientists under intense pressure to meet GCP conditions, only to disappear after recruiting the number of patients necessary for a specific trial, leaving little behind and creating a situation where any trained personnel had to move on. This mode of operation does not support the development of sustainable infrastructure and expertise for clinical trials.

The development of a regulated and sustained clinical trials platform has not been given the attention it deserves in sub-Saharan Africa, despite the urgent present and future need to evaluate the tools being developed to control diseases endemic in the region. There was insignificant participation in product development by African scientists and the lack of well-developed clinical trials platforms in Africa contrasted with the development of strong trial centres, not only in developed countries but also in Asia and Latin America. The regional disparity in capacity development is exemplified by the early development and pre-registration studies of various forms of artemisinin-based combination therapy (ACT) for malaria. These were mostly undertaken in Asia [3,4] with only post-registration studies in children conducted in Africa [5-8]. Consequently, most new anti-malarials have been deployed in Africa with limited knowledge of their efficacy and safety in the local population. The clinical trial centres that have been developed in Africa have generally been strongly linked to northern partners [9] and local scientists not empowered to engage with industry and other partners involved in product development in an equitable and effective way. The few sites that have been developed in this way have tended not to have clinical trials as their primary activity, with little development of a broader research capacity. Most trial sites have been based in or linked to health facilities that were purpose built to provide health care with minimum infrastructure [10], and were thus not equipped to support GCP compliant clinical trials. Missing were appropriate rooms for consenting, monitoring, administration of the study drugs or vaccines and a study participant tracking system.

The relative absence of clinical trial centres in Africa has had negative impact on the development of careers in clinical trials for African clinicians and scientists, and on the development of facilities to conduct GCP and regulatory compliant trials in the region. The human capacity has to be developed to move the clinical trials agenda in Africa to the next level. There is need for a sustained framework to strengthen clinical trial research centres in the sub-Saharan Africa to have a readily available network of centres with the capacity to conduct GCP-compliant clinical trials for the timely evaluation of new products and interventions. Recognition of this need led to the birth of Malaria Clinical Trials Alliance (MCTA) as a programme of the INDEPTH network, to find ways of establishing and sustaining GCP-compliant trial centres in Africa to enable the clinical trials capacity in Africa to begin to match that present in developing countries in Asia and Latin America.

## Case description

### Launch of MCTA

The INDEPTH Network was established in 1998 as a network of demographic surveillance sites in developing countries. One of the early aims was to strengthen the sites in the network to contribute towards the development and evaluation of disease control interventions, including new drugs and vaccines [11]. After several years of development within the network a successful grant application was made to the Bill & Melinda Gates Foundation to support the MCTA, an African-led alliance to strengthen clinical trials capacity and to promote sharing of good practices, with activities linked to ongoing and new clinical trials in malaria. It was set up to take advantage of site-site interactions to share best practices between centres. This was done by identifying centres with expertise in particular areas and facilitating sharing of that expertise with other centres. The main objectives of MCTA were to facilitate the timely development of a network of centres in Africa with the capacity to conduct clinical trials of malaria vaccines and drugs under conditions of GCP; and to support, strengthen and mentor the centres in the network to facilitate their progression towards self-sustaining clinical research centres. To achieve these objectives, MCTA formed strategic partnerships with MMV and MVI, the two leading organizations established to catalyse the development of new anti-malarial drugs and vaccines respectively. The centres invited to join the MCTA platform were from among those previously working with MMV on drug trials and MVI on vaccine trials. The 16 centres comprising the MCTA network are shown in Table 1. MCTA site support focused on strengthening critical capabilities not commonly covered by clinical trial budgets, summarized in Table 2.

**Table 1:** Clinical trial centres comprising the Malaria Clinical Trials Alliance.

Centre	Host Institution	Country
Nanoro Mission Hospital	Institut, de Recherche en Sciences de la Sante/Direction Regionale de I' Ouest	Burkina Faso

Albert Schweitzer Hospital, Lambarene	Medical Research Unit,	Gabon

Kintampo Health Research Centre	Ministry of Health	Ghana

Agogo Hospital, Kumasi Centre for Collaborative Research	School of Medical Sciences Kwame Nkrumah University of Science and Technology (KNUST)	Ghana

Kisumu, Kenya Medical Research Institute (KEMRI)-Walter Reed Project, Kombewa Clinical Trials Unit	Centre for Clinical Research, KEMRI	Kenya

Kisumu, KEMRI-Centres for Disease Control, Siaya District Hospital Clinical Research Centre	Centre for Global Health Research, KEMRI	Kenya

KEMRI-Wellcome Trust Programme, Kilifi	Centre Geographic Medicine Research, KEMRI	Kenya

Ndirande Research Clinic, Ndirande Health Centre	Blantyre Malaria Project, affiliate of Malawi College of Medicine & University of Maryland	Malawi

Lilongwe, Malawi	University of North Carolina Project	Malawi

Manhiça District Hospital	Centro de Investigação em Saúde da Manhiça	Mozambique

Ibadan	Department of Pharmacology, University of Ibadan	Nigeria

State Specialist Hospital, Maiduguri	University of Maiduguri Teaching Hospital	Nigeria

Bagamoyo Research and Training Centre	Ifakara Health Institute	Tanzania

Korogwe, Tanga, Tanzania	Tanga Centre, National Institute of Medical Research	Tanzania

Centre Sur Roi Baudouin	Department of Parasitology, Université Cheikh Anta Diop	Senegal

Fajara	Medical Research Council Laboratories	The Gambia

**Table 2:** Capacity development activities supported by MCTA

Physical Infrastructure Refurbishment	Refurbishment of clinical facilities in hospitals and health centres - providing room and fittings for consenting, out-patient and inpatient monitoring facilities, radiology and pharmacy
	
	Supporting acquisition of critical equipment to ensure study subject safety and study endpoint determination - X-ray equipment and nursing care equipment
	
	Refurbishment of laboratory space and purchase of equipment for clinical chemistry, haematology, malaria diagnosis, bacteriology, sample processing, QC/QA system,
	
	Refurbishment and equipping of data management infrastructure
	
	Refurbishment and equipping clinical trial administration infrastructure
**Capacity development**	Site mentorship by senior scientists who have been involved in site development
	
	Networking of the sites to encourage sharing of good practices and development of collaborations.
	
	Creating visibility of the sites in country and the region by facilitating media interaction

### Procedures

From its inception MCTA set out to work with partners already involved in clinical trials capacity development in Africa, such as AMANET [12] and the Pan-African Bioethics Initiative (PABIN) for GCP and ethics training support, and the Association of Clinical Research Professionals (ACRP) East African Chapter for accreditation of clinical trials personnel. MCTA also took advantage of the existing capabilities within some sites and has facilitated sharing of good practices across the platform. For example, the centre in Kombewa, Kenya had particular skills in malaria diagnosis and microscopy and coordinated the external quality assurance, and there was a pool of trained facilitators supporting the AMANET GCP training based at the various research institutions in Africa. The MCTA site capacity strengthening involved site visits, workshops, inter-site attachment, mentoring, guided infrastructure refurbishment and linking sites to new partners. Care was taken to ensure that for every infrastructure put in place there was expertise to use it and an output expected.

MCTA also draws on the expertise of a team of eminent malaria researchers, who have been involved in site development in Africa, with individual experts linked to particular centres to provide mentorship to the site leadership through site visits. The mentors also advise the secretariat on the capacity and the needs of the site.

### Governance

MCTA is a programme of the INDEPTH network with an autonomous Board of Management and a secretariat operating within the INDEPTH network framework and statutes. The secretariat is responsible for mobilization of funds, site visits, managing the grants, site networking, networking with partners and organizing conferences and workshops. The Board is the final authority that oversees the general governance and approves the annual budget estimates, grants to the sites and guidelines for MCTA activities.

### Achievements

#### Workshops, attachments, programmes and conferences

MCTA has held an annual scientific meeting, with a specific theme each year. This meeting brings together site leaders, mentors and the Board of Management to review the progress made and plans for the future. In addition MCTA has supported workshops targeting areas critical for the conduct of GCP-compliant clinical trials. These are organised jointly with other partners with expertise in these areas to maximize resources utilization. The GCP workshops have been facilitated in collaboration with AMANET and ACRP [13] and malaria diagnostics by the KEMRI-WRP Malaria Diagnostic Centre of Excellence [14]. A summary of the short courses organised to enable staff to acquire the necessary skills to conduct clinical trials and manage their sites effectively is given in Table 3. The workshops have all been over-subscribed. The need for in-depth understanding of GCP principles is made necessary by new requirements of funding agencies for GCP certification of Principal Investigators and study team members.

**Table 3:** Capacity development workshops organised by MCTA

Activity	Attendees (N)			
Year	2006	2007	2008	Total

GCP (number of workshops (n = 7)	61	51	57	189

GCP Certification workshop (n = 1)	0	27	6	33

Malaria diagnosis workshops (n = 5)	14	30	29	71

Strategic Management Process (n = 2)	0	23	25	46

Media workshop (n = 2)	21	20	0	44

MCTA supported the launch of the East African chapter of the ACRP, the first in the region which led to the establishment of accreditation exam centre in Nairobi, Kenya hosted by Strathmore University. Since the inception of the centre in 2007, MCTA has facilitated the accreditation of nine certified clinical research coordinators, three certified research auditors, four certified physician investigators and two GCP facilitators.

MCTA has sponsored four short term cross-site attachments, which have enabled exchange of good practices between the sites and identification common research interests, facilitating the formulation of cross site projects. Five mentors have made visits to seven sites to offer critical unbiased evaluations of site activities. To help centres achieve long-term sustainability, MCTA has organized strategic management workshops facilitated by the Strathmore Business School, Nairobi, to help site teams acquire corporate skills and also develop strategic plans. In order to increase visibility of the centres and narrow the gap in public knowledge on clinical trials, MCTA supported the launch of African Media and Malaria Research Network, coordinated from Accra, Ghana, with local chapters in all the 10 countries where MCTA is supporting sites. This has led to closer collaboration of scientists at the centres and local media, with several sites developing documentaries facilitated by the AMMREN country chapters. Media workshops have been organized to bring together scientists and journalists from the 10 countries to exchange knowledge and develop a common agenda to increase the visibility of malaria research in their countries. The sites have been assisted in the convening of media workshops for their local media to facilitate exchange of ideas and to increase the understanding of the activities of the centres within the media fraternity. AMMREN has facilitated the dissemination of the various activities at the centres in print media and TV channels, including the appearance of MCTA scientists in radio and TV programmes.

MCTA has supported the Malaria Diagnosis Centre of Excellence (MDCoE) in Kisumu to set up a site-based malaria microscopy EQA programme that involves exchange of malaria blood slides between the sites every quarter to monitor the proficiency of the laboratory technologists involved in malaria diagnosis. An accreditation programme for malaria microscopists has also been initiated and so far 28 laboratory personnel have been accredited at basic level and there are plans for advanced level accreditation. In partnership with MDCoE, MCTA has also facilitated the setting up of another centre of excellence in malaria diagnosis at Kintampo, Ghana to increase the number of sites that can offer malaria diagnosis retraining to meet the clinical trials demands. A francophone version the malaria diagnostic training programme will be launched in 2010.

#### Site assessment and refurbishment

The awards of grants for site refurbishment were preceded by an assessment by teams from MCTA or in partnership with MMV and MVI, which identified the needs and strengths of the sites. This was followed by identification of good practices that could be shared across the Alliance. Some of the good practices included health and demographic surveillance (HDSS) for site characterization and study participant follow-up, management of severe malaria, data management, malaria diagnosis and certification of clinical trials professionals. The site visits revealed substantial disparities in the capacities of different centres and even supposedly well-established centres were found to have deficiencies that required urgent support to enable them be fully GCP compliant. This brought to the fore the enormity of the resource needs to have GCP compliant platform meeting the regulatory standards. Between 2006 and 2008, grant applications for capacity strengthening were received from 13 of the 16 MCTA sites and about US$7,300,000 was disbursed for guided site refurbishment, targeting the specific areas critical to conduct of clinical trials (Tables 4 and 5). The difference in amounts is because equipment like X-ray was secured centrally and some of the requested resources were not for physical infrastructure support. The guided infrastructure support has been product development-driven and was performed in consultation with MMV and MVI. The guided support focused on what is critical to the execution of planned activities that are not likely to be covered by the clinical trial budget. The support to the centres was critical for the successful execution of the multi-centre phase III trial of paediatric dispersible artemether/lumefantrine in children [15] and several multi-centre and single centre phase II RTSS malaria vaccine studies [16,17].

**Table 4:** Grants requested and disbursed for guided infrastructure development.

Year	Grants (US$)	
	
	Requested	Approved
2006	2,655,349	1,500,889

2007	5,067,892	3,184,461

2008	3,185,218.75	1,388,273

Total	10,908,459.75	6,073,623

**Table 5:** Grants disbursed for specific activities.

Activity	Year Grants (U$)			
	
	2006 (%)	2007 (%)	2008 (%)	Total (%)
Clinical area refurbishment	214227 (16)	1,215,027 (38)	569850 (22)	1,999,104 (28)

Clinical care equipment	40000 (2)	96,870 (3)	49600 (2)	186,470 (3)

Laboratory refurbishment	322,636 (24)	289,738 (9)	69414 (3)	681,788 (9)

Laboratory equipment	457405 (34)	935750 (29)	506711 (19)	1,899,866 (27)

Administration refurbishment	102700 (8)	409,300 (13)	62626 (2)	574,626 (8)

Administration equipment	214228 (16)	237,776 (8)	128064 (5)	580, 067 (8)

X-ray equipment (11 units)	0	0	1,240,500 (47)	1,240,500 (17)

**Total**	**1,351,195**	**3,184,461**	**2,626,765**	**7,162,421**

The cost of the same equipment varied across countries and this had implications on the funding across the platform. This may call for centralized procurement, but the latter may cause problems with service contracts after installation if local distributors are not involved in the transaction. In some countries equipment did not attract customs duty while in others the duty was high, as much as 50% of the cost of the equipment. In most cases, unless equipment is purchased through a local distributor, after-sales service may be problematic. This was evident when MCTA centrally procured digital X-rays for distribution to the 11 sites involved in the phase III RTS, S malaria vaccine study. The shipment of the equipment from Holland to some countries took several months due to limited flights to some destinations and this was coupled with lengthy custom clearance processes. In some countries, there were no local dealers to provide technical support and reliance on a cross-border technical support wrought with delays, including going through stringent immigration procedures occasioning months of delay to access the site.

The sites conducting malaria drug studies only performed less well over the three years compared to those conducting malaria vaccines or vaccines/drugs. This was partly because there were very few new malaria drugs getting into phase II/III clinical trials compared to malaria vaccines. The performance was based on site evaluation assessment tool that focused on administrative structure, physical infrastructure (lab facilities, clinical facilities, data management, research administration) human resource, site demographic characterization, disease transmission pattern characterization, specific disease management policies, clinical trail SOPs, clinical trial portfolio, collaborations, ongoing projects and capacity for effective collaboration. The three sites that did not receive infrastructure support from MCTA during the three years due to lack of any ongoing or planned clinical trials were all malaria drug trial only sites, however, they participated in staff capacity development workshops supported by MCTA.

### The lessons learned

A summary of the lessons learned in the first three years of operation of MCTA are summarized in Appendix 1. To mitigate some of the challenges identified, partners involved in trial centres as sponsors, collaborators and CROs should not only focus on the short-term gains from their trials, but should work to enhance the capacity of the physical infrastructure, processes and human resource. The centres must give emphasis to building fiscal and legal capacity and systems to enable them negotiate effectively with the partners that need to collaborate with them or use their facilities for academic research or product development. This will enable the centres to leverage resources from the partnerships to support their core and strategic activities to make them more sustainable and competitive. MCTA was a unique programme that supported the development of physical infrastructure. Most funders shy away from this despite knowing how critical it is to sound conduct of research, product development and meeting stringent regulatory standards. The infrastructure development was tightly linked to the development of anti-malarial drugs and vaccines. This ensured that the capacity that was developed was fully utilised not just for clinical trials but to a large extent in improving the health of the community around the centres. The impact on the health of the community is critical in creating a buy in by the community to feel part of the research centre for long term community partnership sustainability. Continued support for clinical trial capacity strengthening is required and can be part of larger initiatives such as malaria eradication/elimination, HIV/TB control, and disease surveillance in resource constrained settings. This continued investment in infrastructure development for clinical trials remains an essential catalyst to seeing Africa fully participate and ultimately be a key player in product development.

It is important to note that developing the appropriate human resource will require as much effort as physical infrastructure development, as the former drives the latter. The human capacity required to support clinical trials is broad (research managers, clinical research coordinators, clinical research auditors, clinical trialists, pharmacologists, immunologists, clinical laboratory technologists, microbiologist, data managers, statisticians, clinical care, epidemiologists & demographers) and a number are needed for optimal execution of a single trial. Developing human resources is not just about supporting the centres but also the wider clinical trials fraternity, other disease control initiatives and ministries of health departments. Well-trained clinical trial personnel are in high demand in the pharmaceutical industry, CROs, medical NGOs and government agencies leading to high turn-over. Sustaining the trial centres with minimal human resources is a recipe for disaster and there must be a concerted effort to plan for staff attrition at all levels. To stem high attrition rates of the critical clinical trial personnel, the partners at each centre must devise innovative ways to keep the staff motivated, such as a clear career path and competitive and attractive remuneration and staff development packages. Human capacity strengthening in the malaria endemic areas will remain crucial to the malaria eradication/elimination agenda to support product and interventions development and policy formulation, implementation, monitoring and evaluation. The rich diversity of the clinical team enables the clinical trial centres at country and regional level to play a central role in partnership with the academic institutions, ministry of health departments and NGOs in the health sector and business schools in facilitating health research human capacity development and the integration of corporate management leadership skills in healthcare.

## Conclusions

The common approach for conducting clinical trials in resource-poor countries has been to do the minimum site development required to perform a specific trial, yet gaps have been identified in the clinical trials capacity. This has impeded sustainable development and local ownership of the research agenda at sites and has not assisted their progression to viable research centres. MCTA has, in a short time, been able to provide the sites in the alliance more flexibility and ownership. This has reduced the lead time necessary for the execution of clinical trials at these centres. The current number of trained and qualified clinical trialists and support staff in Africa is still small and in dire need for expansion, through the provision of long-term training at graduate, postgraduate and professional levels. This is an expensive but necessary venture and must be addressed by all interested partners on a sustainable basis. Those involved in building a sustainable functional clinical research platform in Africa should bring on board African governments, private sector, bilateral and multilateral agencies and philanthropic foundations. All these partners are stakeholders in the activities at the research centres in different capacities at different times. Concerted support will succeed if it is based on trust and guided by professionals who understand the dynamics of clinical research in resource-poor settings.

## Abbreviations

AMANET: African Malaria Network Trust; AMMREN: African Media and Malaria Research Network; ACRP: Association of Clinical Research Professionals; BoM: Board of Management; CRC: Clinical Research Coordinator; CCRC: Certified Clinical Research Coordinator; CDC: Centres for Disease Control & Prevention; CRA: Clinical Research Associate; CRO: Contract Research Organization; DNDi: Drugs for Neglected Disease Initiative; EDCTP: European & Developing Countries Clinical Trials Partnership; EMVI: European Malaria Vaccine Initiative; EQA: External Quality Assurance Scheme; FIND: Foundation for innovative New Diagnostics; INDEPTH: International Network for Demographic Evaluation of Populations and their Health in developing countries; KEMRI: Kenya Medical Research Institute; HDSS: Health & Demographic Surveillance System; HIV: Human Immunodeficiency Virus; GCLP: Good Clinical and Laboratory Practice; GCP: Good Clinical Practice; MCTA: Malaria Clinical Trials Alliance; MDCoE: Malaria Diagnosis Centre of Excellence; MIM: Multilateral Initiative on Malaria; MMV: Medicines for Malaria Venture; PABIN: Pan-African Bioethics Initiative; PATH-MVI- Program for Appropriate Technology in Health-Malaria Vaccination Initiative; QA: Quality Assurance; QC: Quality Control; TDR: WHO: Tropical Disease Research program; TB: Tuberculosis; UNC: University of North Carolina, Chapel Hill; WRP: Walter Reed Project

## Competing interests

The authors declare that they have no competing interests.

## Authors' contributions

BO is the Senior Clinical Trialist with MCTA program; designed and coordinated the writing of the manuscript, RB is a Clinical Trialist with MCTA; contributed to the design and writing of the manuscript, DD was a Clinical Trialist with MCTA and contributed to the writing of the manuscript, PGS is mentor with the MCTA and contributed to the writing of the manuscript and FNB is the MCTA Programme Director and had overall oversight on the design and writing of the manuscript. All the authors reviewed the final manuscript and approved it before submission.

## Appendix 1. Lessons learned through the first 3 years of operation of MCTA

### Physical infrastructure

1. Currently available clinical facilities were largely developed for clinical care and the very few developed for clinical trials were designed for small studies and were not suitable for medium to large studies (in terms of space for recruitment, consenting, screening, laboratory evaluations) and had no provision for equipment back up or calibration, service contracts and QC/QA programmes.

2. It was possible to upgrade capacity at sites with relatively modest levels of funding and less the US $2 million that been recommended to refurbish a site to perform clinical trials to GCP standards [9]. The sites have developed a feeling of ownership as the support has not been linked to a specific product or partner.

### Capacity

1. Linking of capacity strengthening to specific intervention trials at the sites is essential to avoid mismatch with resultant redundant infrastructure

2. Frequent GCP, GCLP, malaria diagnosis and human protection training programmes are required due to staff turn over, expansion, maintenance of accreditation and also to satisfy regulatory requirements for retraining.

3. There is need for centralized advice on availability, selection, ordering, installation and post-installation service of equipment. However, to ensure optimal acquisition and maintenance of equipment, the supply of equipment to the sites was done by the site teams through local distributors based in country or in a nearby country. This ensured that there was continued technical support and supply of specific consumables after installation. The X-ray machines were the only equipment procured centrally, but MCTA ensured that there was regional or in country support post installation.

4. Sharing of information across the network and having support readily available to assist other sites in their areas of competence has been very valuable.

5. The quality of funding requests improved as the site teams engaged better with the secretariat with a focus on plans for sustainability

6. There is need for sites to have clear channels of communication with regulatory authorities, independent review boards and relevant government departments, whose activities have a direct bearing on the site operations. Failure to establish these channels can result in delays in approvals for trial conduct and equipment acquisition.

7. The long term development and sustainability of sites/centres will require diversification of their research portfolio [18] within a specific disease and across diseases. Capacity for research, other than through clinical trials, needs to be developed as it is unlikely that a site can be sustained in the long term only through the conduct of clinical trials.

8. Some of the sites conducting malaria research were among the first to register a reduction in malaria burden [19], which exemplifies the indirect benefit of research to the community by improved bed-net coverage, better uptake of new intervention strategies i.e. health education and effective drugs.

9. The changing transmission dynamics of malaria may result in some sites becoming unsuitable for evaluating new malaria interventions, because of a low malaria incidence. However the capacity developed at the sites should enable them to provide platforms for monitoring the changing epidemiology and act as sentinel sites for malaria resurgence. There will be a need to identify and strengthen new sites in areas with higher disease burden, making site development and sustenance a continuous process.

10. Even with the capacity development provided through MCTA, there is limited number of personnel with expertise in clinical trials across all the sites to meet the likely demands for evaluating new malaria interventions [18]. A long-term training framework for future clinical trialists is required to be able to achieve a critical mass of trialists at the sites and in the region.
